# CFRNet: Cross-Attention-Based Fusion and Refinement Network for Enhanced RGB-T Salient Object Detection

**DOI:** 10.3390/s24227146

**Published:** 2024-11-07

**Authors:** Biao Deng, Di Liu, Yang Cao, Hong Liu, Zhiguo Yan, Hu Chen

**Affiliations:** 1 Dongfang Electric Autocontrol Engineering Co., Ltd., Deyang 618000, China; dengbiao9163@dongfang.com; 2 College of Computer Science, Sichuan University, Chengdu 610000, China; liudi007@stu.scu.edu.cn (D.L.); caoyang@stu.scu.edu.cn (Y.C.); huchen@scu.edu.cn (H.C.)

**Keywords:** RGB-T salient object detection, RGB–thermal fusion, cross-attention, fusion and refinement

## Abstract

Existing deep learning-based RGB-T salient object detection methods often struggle with effectively fusing RGB and thermal features. Therefore, obtaining high-quality features and fully integrating these two modalities are central research focuses. We developed an illumination prior-based coefficient predictor (MICP) to determine optimal interaction weights. We then designed a saliency-guided encoder (SG Encoder) to extract multi-scale thermal features incorporating saliency information. The SG Encoder guides the extraction of thermal features by leveraging their correlation with RGB features, particularly those with strong semantic relationships to salient object detection tasks. Finally, we employed a Cross-attention-based Fusion and Refinement Module (CrossFRM) to refine the fused features. The robust thermal features help refine the spatial focus of the fused features, aligning them more closely with salient objects. Experimental results demonstrate that our proposed approach can more accurately locate salient objects, significantly improving performance compared to 11 state-of-the-art methods.

## 1. Introduction

Salient object detection (SOD) is a visual attention-related task that aims to locate the most significant information in visual scenes, consistent with the human visual system [1,2]. SOD can eliminate redundant information in computer vision tasks and improve model performance, as the applying of visual attention in preprocessing steps is prevalent for highlighting areas of interest. Thus, SOD advancements benefit various tasks, such as image editing and segmentation [3,4,5], person re-identification [6,7], and neural architecture search [8]. These tasks achieve this through the visible information typically contained in RGB images; hence, they are referred to as RGB SOD.

While RGB SOD methods excel under optimal conditions [1,9], they face challenges in low-light or low-contrast environments. In these scenarios, the reliance on visible information can lead to overlooking crucial areas with limited visibility.

To address these challenging scenes, we build upon the concept introduced by Zhang et al. [10], who demonstrated the use of additional modalities to enhance performance in situations where RGB images alone are insufficient. Thermal images can better present the overall shape of objects in various complex scenarios [11], and the higher the temperature of the object, the stronger the infrared radiation, making the object more prominent in thermal images. Thus, a novel SOD branch called RGB-T SOD has emerged, and many attempts have been made, with a considerable number of them showing promise in resisting the impact of harsh environments [12,13].

Several recent CNN-based models show appreciable performance [14,15,16,17], and their ability to gather local information from neighboring pixels and build large perceptual fields plays a significant role.

However, these methods focus on modality fusion but rarely explore the relationship between RGB and thermal images. Firstly, RGB and thermal images contain different pieces of useful information in various situations, and the dynamic modal weight should be considered to prioritize the modality that provides more useful knowledge. Secondly, RGB and thermal images display visual and thermal information, but they may highlight different objects. For instance, salient objects may not be hotter than the background, and the background area may display more heat, causing the model to focus on non-salient areas. Additionally, the introduction of thermal imaging aims to provide more information, but whether thermal information is fully utilized has not received sufficient attention.

Considering the first question, when the scene is too dark or too bright, visible information is limited, and thermal information becomes more critical. Conversely, under normal illumination, visible information is the primary source for identifying salient objects. Therefore, we evaluate the effects of different modalities based on the scene’s lighting conditions. Regarding modality fusion, it is essential to ensure that both modalities highlight the same object. It is optimal to adjust thermal features using visual features, as the definition of salient objects is derived from visual attention areas. Additionally, we need an effective method to identify and compensate for the deficiencies in the fused features using thermal information. Thus, we should find the similarities between the fusion features and thermal features.

To tackle the aforementioned issues, we first propose a Modality Interaction Coefficient Prediction (MICP) module based on an illumination prior, which predicts a coefficient representing the importance of the two modalities and determines the modality weight for fusion. This method introduces an illumination prior calculated using traditional formulas, which prior is concatenated with the original images to enrich them with brightness. A lightweight neural network is then used to process these images and obtain the weights, thereby determining the corresponding thermal weights.

For adjusting the thermal features, we propose a thermal adjustment method to guide the thermal encoder to focus more on salient regions in RGB features. The guidance information consists of RGB features from the final layer and RGB features from the same stage as the current thermal stage. This guidance uses the final RGB features because they contain rich semantic knowledge, while spatial details are lost in downsampling operations, which also explains why we introduced RGB information from the current layer. The combination of these features provides overall position and specific details of salient objects in RGB, allowing thermal features to gradually focus on visually salient areas.

When we fuse the features, a module named Cross-attention-based Fusion and Refinement Module (CrossFRM) is used to fuse multi-modal features from each layer. The features of the two modalities share the same saliency and represent different viewpoints of the scene. We fuse the features using a coefficient. Then, we perform feature fusion between groups to obtain the final fusion features. After fusing, the proposed Swin-based Cross-attention module (Swin-Cross) is used to refine the fusion features. Cross-attention calculates the focus of one feature on another, allowing us to identify thermal feature content that the fusion features may not emphasize. We then compensate for these areas in the fusion feature, ensuring that the fused feature contains sufficient visible and thermal information without any deficiencies. In the decoding stage, we use the decoding results of the previous layer and the fusion features of the current layer to obtain the final decoding results and aggregate multi-scale salient information.

To address these challenges, we propose CrossRefine, a novel network for RGB-T SOD that optimizes multi-modal data utilization. Our approach yields significant improvements:

- The MICP module achieves dynamic modality weighting, improving mean F-measure by 2% on the VT821 dataset compared to static weighting methods.
- Our thermal adjustment method enhances the alignment of thermal features with salient objects, increasing the structure measure by 2.6% on the VT821 dataset.
- CrossFRM effectively refines fused features, achieving an average 7.9% reduction in MAE across the VT821, VT1000, and VT5000 datasets.

Our work addresses these challenges through the following key contributions:

- We propose a Modality Interaction Coefficient Prediction (MICP) module that dynamically determines feature fusion weights based on scene illumination, enhancing the model’s adaptability to various lighting conditions.
- We introduce a novel thermal adjustment method that guides the thermal encoder to focus on visually salient objects by incorporating RGB saliency information.
- We develop a Cross-attention-based Fusion and Refinement Module (CrossFRM) that refines fused features using thermal information, ensuring comprehensive integration of visible and thermal data.
- Extensive experiments on three benchmark datasets demonstrate that our approach significantly outperforms 11 state-of-the-art methods in RGB-T salient object detection.

The rest of this article is organized as follows: Section 2 introduces the related work on RGB and RGB-T SOD. Section 3 provides a detailed description of our method. Section 4 presents the experimental results and analysis. Finally, Section 5 concludes the paper with a summary of our findings.

## 2. Related Work

### 2.1. RGB-T Salient Object Detection

Thermal images can capture the heat emitted by objects and are insensitive to variations in illumination and weather, making them suitable for handling scenes captured under adverse conditions. Therefore, thermal imaging is a promising supplement to RGB images for SOD.

Recent advancements in deep learning have significantly contributed to RGB-T SOD. Notable works include universal datasets and models by Wang et al. [15] and Tu et al. [16,17], and Zhang et al.’s [18] comprehensive approach involving multi-level feature extraction and multi-modal fusion. Tu et al. [19] proposed a multi-interactive dual-decoder to integrate the multi-level interactions of dual modalities and global contexts. Gao et al. [20] simulated visual color stage doctrine to fuse cross-modal features in stages and designed a bi-directional multi-scale decoder to capture both local and global information. Wang et al. [21] adopted the guidance manner of one modality over the other to fuse the two modalities. Song et al. [22] proposed a method for completing multi-modal fusion using multi-graph affinity interaction. Wang et al. [23] proposed a thermal sensing early fusion network for cross-brightness salient object detection, which is trained on normal-lighting images and performs salient object detection on low-lighting images.

As Transformers drive the development of computer vision, cross-attention mechanisms are widely used in multi-modal tasks.

### 2.2. Attention-Based Model

Convolution-based attention is widely used in many computer vision tasks. In SOD tasks, researchers [24,25,26,27] apply convolution-based attention to obtain attention information that is closely related to salient information.

Research on Transformers has become mainstream in the field of attention research. Dosovitskiy et al. [28] proposed the ViT model, which applies Transformers to image classification and has become a milestone in computer vision due to its simple architecture, significant performance, and strong scalability. However, ViT lacks multi-scale visual feature extraction and involves a large number of parameters and computations. Liu et al. [29] proposed the Swin Transformer, a sliding window mechanism-based approach. Compared to ViT, it can extract local information from images and has stronger recognition abilities for targets of different scales. Zheng et al. [30] pioneered the application of Transformers to the segmentation field, using ViT [28] to extract features and building a lightweight convolutional decoder to obtain predictions. Chen et al. [31] proposed CrossViT to address the problem of multi-scale information loss in ViT. After calculating *Q*, *K*, and *V* separately, the two branches complete the calculation of cross-attention, thus enabling the interaction of the two types of scale information.

## 3. Method

### 3.1. Architecture Overview

**Figure 1:**
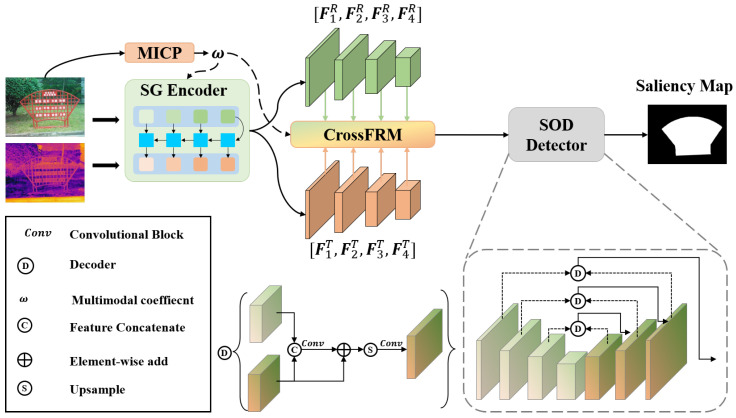
CrossRefine architecture overview: integrating MICP, SG Encoder, and CrossFRM for enhanced RGB-T salient object detection.

In this paper, our main objective is to identify underutilized segments within thermal information by leveraging already fused features. This enables the integration of these segments into the existing features, thereby refining the fusion of thermal and RGB features. To achieve this, we propose a dual-branch encoder–decoder network structure named CrossRefine for RGB-T SOD, as illustrated in Figure 1. We first input the RGB image into the Modality Interaction Coefficient Prediction (MICP) module to obtain the modality weight *w*, which is used to weigh the fusion of RGB and thermal features. We utilize ResNet34 [32] as the encoder for RGB features, removing the last pooling layer and fully connected layers for classification, and a ResNet34 [32] with the same modifications to encode thermal features. The multi-level RGB and thermal features are denoted as $F_{i}^{R}$ and $F_{i}^{T}$, respectively, where i ranges from 1 to 4. This notation represents four distinct levels of feature extraction for each image modality. To address the challenge of non-common saliency phenomena and effectively integrate RGB and thermal features, we develop a thermal adjustment method that inputs weighted visible spatial information into thermal features at each encoder stage. This approach gradually adjusts the focus of thermal features, emphasizing regions more salient in the RGB images. During the fusion process, the RGB and thermal features are initially fused using the multi-modal interaction coefficient α. Subsequently, the fused features, along with the thermal features, are input into the Swin-based Cross-attention module (Swin-Cross) to identify areas where fusion features resemble thermal features. These similar parts indicate useful related information, and these regions are refined with additional thermal information extracted from those parts. In the decoding stage, we adopt a single-branch CNN that includes upsampling layers to decode fused features and predict the saliency map.

### 3.2. Modality Interaction Coefficient Prediction Module

**Figure 2:**
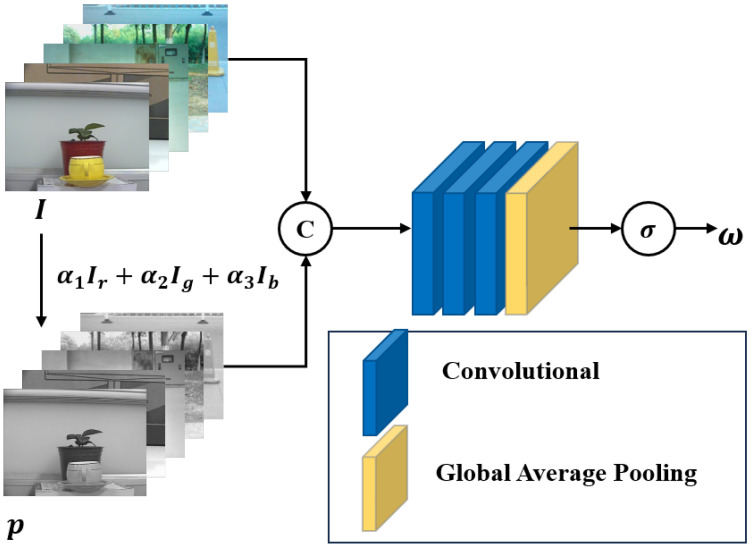
MICP module: modality interaction coefficient extraction using illumination prior. Note: $\alpha_{1}$, $\alpha_{2}$, $\alpha_{3}$ represent the weight coefficients used in converting the RGB color image to a grayscale image.

Based on the definition of salient objects, it is evident that salient object detection primarily relies on visible information, with auxiliary modalities providing additional knowledge to enhance task performance and model robustness. In RGB-T SOD, RGB images remain prominent while thermal images provide supplementary information. However, as discussed above, the importance of each modality is clearly scene-dependent, and there is no universal weighting method suitable for all scenarios. Thus, obtaining dynamic weights to control the balance between RGB features and thermal information in the multi-modal fusion features of our proposed CrossRefine is essential. This ensures that the fusion features are optimally adapted to the target scene. To this end, we design a Modality Interaction Coefficient Prediction (MICP) module to predict modality coefficients as modality weights for controlling multi-modal interaction during fusion stages, as shown in Figure 2.

According to the analysis above, we explore methods to introduce illumination information to adjust modality weights. We incorporate an illumination prior [33] into the original RGB image and extract the illumination map using an illumination estimator, which can be formulated as follows:

(1) $$I_{m}=E\left(\left[I,p\right],\theta \right)$$

Here, $I_{m}$ represents the illumination map, *I* is the input RGB image, p denotes the illumination prior, E refers to the illumination estimator from [33], and θ represents the parameters of E. Additionally, [·,·] denotes a channel-wise concatenation operation. The illumination map clearly indicates whether the image is captured in low-light or normal-lighting conditions.

Our proposal aims to obtain a weighting coefficient for assessing RGB and thermal features in different scenes, rather than using an illumination map to represent illumination details pixel by pixel. By considering the approach from a local to a global perspective, we recognize that the illumination values of all pixels fluctuate around the global average illumination value, which closely approximates the overall illumination level of the scene. Excessive focus on the details inherent in the scene illumination map may cause features to concentrate on specific pixels, leading to overfitting. Therefore, we employ global average pooling to obtain the average value of the scene illumination map, and we predict the coefficient for multi-modal interaction. We append a sigmoid activation function to regulate the coefficient to a range of 0 to 1, serving as the modality weight. The formula is as follows:

(2) $$\omega =\sigma (Conv\left(\mathrm{GAP}\left(I_{m}\right)\right))$$

Here, ω is the predicted coefficient, Conv(·) represents a convolution block used to predict coefficients, GAP(·) computes the global average value, and σ denotes the sigmoid activation function.

The existing modality interaction coefficient ω will be used to weigh RGB features in subsequent modules. It represents the proportion of contribution from visible information in the fused features. As the value approaches 1, the influence of RGB features becomes more significant, while the impact of thermal features decreases. In the thermal encoding stage, we use ω to control the amount of guidance that includes semantic and detailed information from visible data, avoiding low-quality information that reduces performance. Further details will be introduced in Section 3.3.

### 3.3. Saliency-Guided Encoder

**Figure 3:**
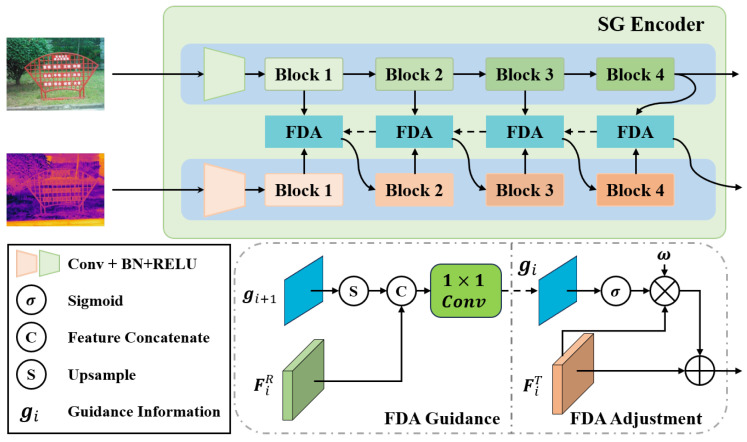
SG Encoder with Feature Dynamic Adjustment (FDA) module: enhancing thermal and visible feature integration.

Thermal images can serve as an effective source of auxiliary information, but directly fusing separately processed thermal and visible information is unreasonable due to the significant differences in the spatial regions highlighted by these representations. Thermal information is not strictly correlated with visible information, nor does it consistently align with salient objects. As shown in Figure 3, the Saliency-Guided (SG) Encoder integrates thermal and visible information using the Feature Dynamic Adjustment (FDA) module, which progressively shifts the focus of thermal features toward visually salient targets.

Based on the analysis and inference, our intuition is to introduce visible information into thermal information and adjust the thermal features to ensure that their prominent parts are similar to RGB features, indirectly establishing a correlation between thermal information and salient objects. Therefore, we investigate a thermal adjustment method during the thermal encoding stages to achieve this goal, allowing subsequent thermal encoders to progressively shift the focus of thermal features towards visually salient targets.

It is common knowledge that deeper features possess more abundant semantics, which are crucial for precisely locating salient objects. Meanwhile, the detailed information within shallower features helps address edge details. Therefore, we utilize the deepest embedded features to extract corresponding parts from shallow features and integrate them into the thermal features in a Feature Dynamic Adjustment (FDA) module. The adjustment method is as follows:

(3a) $$\begin{array}{cc} g_{i} & =\left\{\begin{array}{cc} Conv_{1\times 1}\left(F_{i}^{R}\right), & i=4\\ Conv_{1\times 1}\left(\left[UP\left(g_{i+1}\right),F_{i}^{R}\right]\right), & i=1,2,3 \end{array}\right. \end{array}$$

(3b) $$\begin{array}{cc} {\hat{F}}_{i}^{T} & =F_{i}^{T}+\omega \cdot \sigma \left(g_{i}\right)\cdot F_{i}^{T},i=\left\{1,2,3,4\right\} \end{array}$$

Here, $g_{i}$ represents the guidance information, ${\hat{F}}_{i}^{T}$ denotes the adjusted thermal features of the *i*th layer, and ω is the modality interaction coefficient predicted by MICP.

As the equation shows, we first obtain the guidance information of $F_{4}^{R}$ as $g_{4}$, then gradually derive the guidance information $g_{i}$ using deeper guidance and corresponding RGB features, and multiply it with the corresponding thermal features $F_{i}^{T}$, i = {1, 2, 3, 4} to adjust the thermal features. In low-light conditions, information from RGB features is less reliable, and useful information primarily comes from thermal features. Therefore, in such circumstances, thermal supplementation guided by visible semantics should be minimized. In contrast, when the scene illumination is sufficient, RGB features are reliable, and thermal supplementation should be maximized. To address these situations, it is necessary to adopt a weighted adjustment strategy, using the output of MICP for weighting. This approach allows us to maintain thermal features, optimally utilize high-quality RGB features in sufficient illumination, and minimize the impact of low-quality RGB features in poor light.

### 3.4. Cross-Attention-Based Fusion and Refinement Module

After the adjustment of thermal features is completed, the differences in representation content between the two modalities are reduced, allowing each modality to provide information about salient objects. It is necessary to fuse these two modal features to obtain a fusion feature with sufficient representation capacity for salient objects. However, the fusion quality of multi-modal features remains a topic worth exploring.

**Figure 4:**
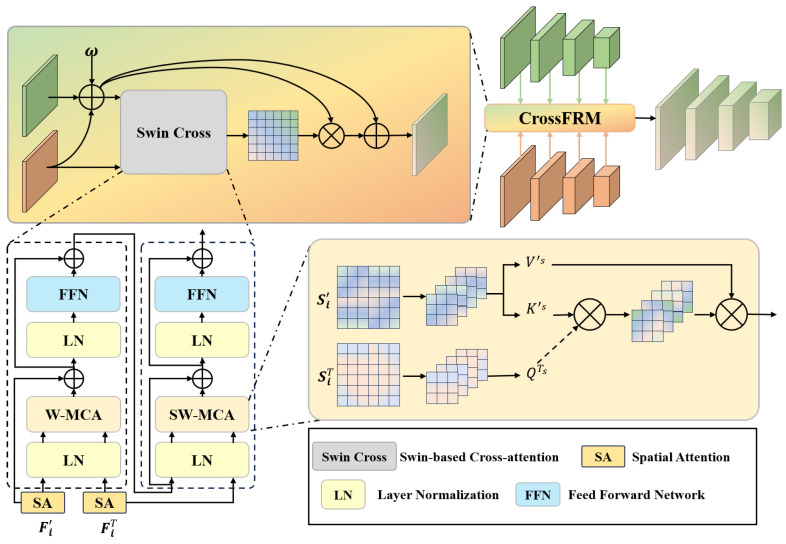
CrossFRM: multi-modal feature fusion and refinement.

To address the weak correlation between thermal features and salient objects in RGB-T SOD, we leverage RGB features to guide thermal feature refinement. This approach integrates semantic and textural information from RGB data to align thermal features with visually salient regions. However, the gap between thermal information and salient objects remains unaddressed, leading to deviations in the quality of fusion and whether the fusion feature fully exploits the thermal information. To address this issue, we propose an approach to explore the parts of the thermal features that the fusion features focus on. The main strategy is to identify the regions of interest in the thermal features, conduct an in-depth exploration of these regions for underutilized information, and extract this information into the fusion features to refine them. Specifically, we propose a Cross-attention-based Fusion and Refinement Module (CrossFRM) to fuse RGB and thermal features and refine the fusion features using thermal information. The module is shown in Figure 4.

First, we fuse the two features based on the coefficient ω to obtain the fusion feature $F_{i}^{\prime }$. Then, we adopt the Swin-based Cross-attention module (Swin-Cross) to interact with the focus of thermal features and fusion features. The adjusted thermal features are closer to the salient objects, effectively achieving target localization and boundary distinction. By utilizing the spatial information of thermal features, it is possible to explore more similar parts of the attention content. This is formally manifested as the interaction between the two spatial attention results. Finally, we use the refined focus of fusion features to adjust and further refine the fusion features. The module can be formulated as follows:

(4a) $$\begin{array}{cc} S_{i}^{\prime /T} & =SA(F_{i}^{\prime /T}),i=1,2,3,4 \end{array}$$

(4b) $$\begin{array}{cc} F_{i}^{\prime \prime } & =F_{i}^{\prime }\cdot \left(1+SC\left(S_{i}^{\prime },S_{i}^{T}\right)\right),i=1,2,3,4 \end{array}$$

Here, $F_{i}^{\prime /T}$ means $F_{i}^{\prime }$ and $F_{i}^{T}$; the same applies to $S_{i}^{\prime /T}$, and $F_{i}^{\prime }$ and $F_{i}^{T}$ denote the fusion features and thermal features in the $i^{th}$ layer, respectively. SA(·) computes spatial attention, and SC(·) denotes the Swin-Cross module. The refined features are represented by $F_{i}^{\prime \prime }$.

In Swin-Cross, the interaction between the spatial information of fusion features and thermal features is conducted as follows:

(5a) $$\begin{array}{cc} Y_{i} & =Softmax\left(\frac{Q_{i}^{T}{K_{i}^{\prime }}^{⊤}}{\sqrt{d}}\right)V_{i}^{\prime },i=1,2,3,4 \end{array}$$

(5b) $$\begin{array}{cc} S_{i}^{\prime \prime } & =S_{i}^{\prime }+Y_{i}+FFN\left(Norm\left(S_{i}^{\prime }+Y_{i}\right)\right),i=1,2,3,4 \end{array}$$

Here, $Q_{i}^{T}$, $K_{i}^{\prime \prime }$, and $V_{i}^{\prime }$ represent the query, key, and value, respectively. FFN(·) denotes the feed-forward network. The superscript T in $K_{i}^{\prime ⊤}$ denotes the transpose operation. This interaction is formally manifested as the interaction between the two spatial attention results. Then, we use the refined attention of fusion features to adjust and further refine the fusion features.

After obtaining the refined features, the multi-scale fusion features are input into the decoder, and we generate the multi-scale saliency maps $M_{i}$ using 1×1 convolutional blocks.

For the loss function *L*, we employ a combination of BCE loss $L_{bce}$ and Soft-IoU loss $L_{iou}$:

(6) $$L=L_{bce}+L_{iou}$$

Here, $L_{iou}$ and $L_{bce}$ denote the binary cross-entropy loss and the soft intersection over union loss, respectively.

## 4. Experiments

### 4.1. Datasets

To validate the proposed model and components, we evaluate our method and all compared methods on three public benchmark datasets: VT821, VT1000, and VT5000. VT821 contains 821 pairs of RGB images, thermal images, and saliency maps across 11 challenging scenes, with many images being defective. VT1000 includes 1000 image pairs in 10 challenging scenes, with fewer defective images. VT5000 comprises 5000 image pairs across 11 challenging scenes, featuring higher image quality and greater detection difficulty. VT821 contains many defective samples due to manual alignment issues, resulting in unnatural noise and black padding. VT1000 lacks samples from adverse weather conditions, making it less challenging overall. VT5000 offers the most diverse range of scenarios with higher image quality and detection difficulty. To balance comprehensiveness and conciseness, we conducted a comprehensive evaluation on all datasets (see Table 1 and Table 2) while selecting [VT821] for in-depth analysis (see Table 3 and Table 4).

### 4.2. Evaluation Metrics

To fully demonstrate the performance differences between methods, we adopt four metrics to quantitatively evaluate the models. S-measure ($S_{m}$) focuses on region-aware and object-aware structural similarities between the saliency map and the ground truth. MAE is the mean absolute pixel error. F-measure ($F_{m}$) is a metric about region-based similarity based on precision and recall. E-measure ($E_{m}$) characterizes both image-level and local pixel matching. In this paper, we use adaptive $F_{m}$ and adaptive $E_{m}$, which adopt twice the average as the threshold.

### 4.3. Implementation Detail

We implement our model in PyTorch (version 1.8.1) using a single NVIDIA Tesla P100 GPU (manufactured by NVIDIA Corporation, Santa Clara, CA, USA). The CPU architecture is PPC64el. The backbone network is initialized with weights pretrained on ImageNet (ILSVRC2012), while the other modules are initialized randomly. We adopt the AdamW optimizer for training. The total number of training epochs is set to 300, and the initial learning rate is $5\times 10^{-4}$, which decays by a factor of 0.1 at the 50th epoch and by 0.01 at the 100th epoch. All images in the training set are cropped randomly, and those in the testing set are resized to 352 × 352.

### 4.4. Comparison with SOTA Methods

Our proposed CrossRefine is compared with 11 state-of-the-art (SOTA) models, including ADFNet, CGNet, CSRNet, MMNet, MIDD, ECFFNet, APNet, DCNet, LSNet, TAGFNet, and MGAI. To ensure the fairness of the comparison results, the saliency maps for evaluation are all provided by the authors.

**Figure 5:**
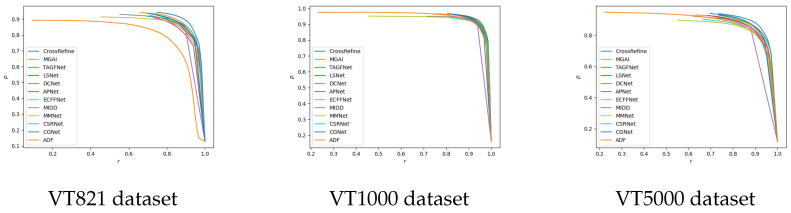
Precision–recall curve comparison for different methods on VT datasets.

Table 5 shows the comparison results of the four metrics. Compared to other methods, our model achieves excellent performance in almost all metrics on the VT821, VT1000, and VT5000 datasets. This improvement is largely due to our proposed fusion and refinement module and design. Taking the VT821 dataset as an example, the adaptive F-measure, adaptive E-measure, and MAE improve by 0.59%, 0.54%, and 3.22%, respectively. The PR curve also verifies the effectiveness and advantages of our proposed method (see Figure 5 for the precision-recall curve comparison).

**Figure 6:**
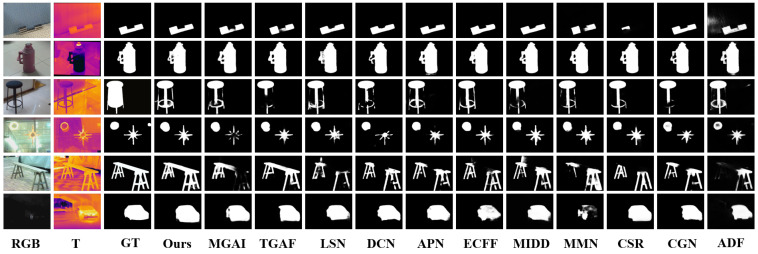
Qualitative comparison of salient object detection models on RGB-T images: visualization of saliency maps. Note: The images show saliency maps produced by various models, where brighter regions indicate higher saliency. These maps highlight the most visually important objects in the scene as determined by each model, rather than representing semantic segmentation results.

To make qualitative comparisons, we present some visual examples in Figure 6. While the output of our method may visually resemble semantic segmentation results, it is important to note that CFRNet produces saliency maps indicating object importance, not semantic class labels. It can be observed that our method achieves better detection results than other methods in several challenging cases, and it is evident that the model performs well under varying illumination conditions. The 3rd row demonstrates that our method can perform well even when the sample is mislabeled. All the results indicate that our approach can better adapt to different scenes and perform effectively through cross-modality fusion.

### 4.5. Ablation Studies

We conduct ablation studies on RGB-T SOD to verify all components.

(1) *The effectiveness of MICP:* MICP is a significant component in our model for obtaining dynamic, illumination-aware fusion features. While the model can implicitly learn information related to environmental illumination, it cannot guarantee the acquisition of correct knowledge. Therefore, we conducted experiments on MICP to explore its effectiveness and to determine whether introducing brightness priors is necessary. The results are shown in Table 1. From the results, it can be seen that explicitly obtaining a multi-modal interaction coefficient allows the network to learn relevant information, which is beneficial for improving the detection performance of the model. The introduction of brightness priors in MICP further achieved better performance indicators without involving an increase in model parameters. Therefore, it can be inferred that the performance improvement directly comes from the information provided by the brightness priors themselves.

(2) *The effectiveness of FDA:* The most important component in the SG Encoder is the FDA, where dynamic weight is introduced while adjusting the thermal features with guidance information. To determine the best existing adjustment method, we conducted experiments, including the acquisition method of guidance information. The experimental results are shown in Table 3. It can be seen that the dynamic adjustment of thermal infrared features can effectively enhance the attention of fusion features to salient targets and improve performance. Meanwhile, by gradually integrating deeper guidance information with corresponding level features, semantic and detailed content can be effectively combined to obtain better guidance information. Our methods perform better than direct guidance using a 1 × 1 convolutional block, upsampling $g_{4}$, and concatenating the features with the result of upsampling $g_{4}$ ($g_{4}$ upsample + concatenate), as shown in Table 4.

(3) *The effectiveness of CrossFRM:* We remove the Swin-Cross module to demonstrate the effectiveness of refinement. From Table 2, we observe that the use of Swin-Cross leads to performance improvement. Additionally, a window-based cross-attention module performs better than a traditional cross-attention block, which further proves that window-based attention is effective in processing image features that focus on local information. The results in Table 2 also indicate that the current number of attention layers is optimal for achieving the best performance of the model, while adding more layers does not result in further performance improvement.

## 5. Conclusions

In this article, we proposed a fusion-based refinement method for RGB-T salient object detection and designed a corresponding network. In this network, a Swin-based cross-attention mechanism is used for spatial information exchange. By combining Swin-based cross-attention with convolutional neural networks, both global and local information is fully captured. The multi-modal interaction coefficient prediction module introduces a brightness prior to constrain dynamic weight acquisition, and the saliency-guided encoder uses RGB feature information to establish the correlation between thermal features and salient objects. In the refinement process, the robust spatial information of thermal features is fully utilized. CrossRefine achieves state-of-the-art (SOTA) performance and demonstrates good results on public datasets. We aim to further improve performance in the future and explore lightweight-related topics.

## Figures and Tables

**Table 1:** Ablation study results of MICP and brightness prior on VT datasets.

MICP	Bright Prior	VT821				VT1000				VT5000			
		MAE↓	$F_{m}^{\mathrm{adap}}↑$	$S_{m}↑$	$E_{m}^{\mathrm{adap}}↑$	MAE↓	$F_{m}^{\mathrm{adap}}↑$	$S_{m}↑$	$E_{m}^{\mathrm{adap}}↑$	MAE↓	$F_{m}^{\mathrm{adap}}↑$	$S_{m}↑$	$E_{m}^{\mathrm{adap}}↑$
		0.034	0.828	0.875	0.913	0.021	0.895	0.924	0.952	0.033	0.839	0.882	0.925
✓		0.032	0.836	0.873	0.914	0.021	0.899	0.926	0.955	0.032	0.845	0.884	0.927
✓	✓	**0.030**	**0.845**	**0.885**	**0.925**	**0.020**	**0.900**	**0.926**	**0.955**	**0.032**	**0.846**	**0.885**	**0.928**

**Table 2:** Ablation study results of Swin-Cross and refinement methods on VT datasets.

Refinement Method	VT821				VT1000				VT5000			
	MAE↓	$F_{m}^{\mathrm{adap}}↑$	$S_{m}↑$	$E_{m}^{\mathrm{adap}}↑$	MAE↓	$F_{m}^{\mathrm{adap}}↑$	$S_{m}↑$	$E_{m}^{\mathrm{adap}}↑$	MAE↓	$F_{m}^{\mathrm{adap}}↑$	$S_{m}↑$	$E_{m}^{\mathrm{adap}}↑$
No Refinement	0.033	0.831	0.877	0.914	0.022	0.893	0.920	0.949	0.034	0.841	0.877	0.918
Spatial Attention	0.031	0.839	0.880	0.920	0.021	0.893	0.923	0.952	**0.032**	0.842	0.882	0.922
Spatial Attention + Cross-Attention	0.032	0.836	0.881	0.924	0.021	0.897	0.924	0.953	0.033	**0.846**	0.884	**0.928**
**CrossFRM**	**0.030**	**0.845**	**0.885**	**0.925**	**0.020**	**0.900**	**0.926**	**0.955**	**0.032**	**0.846**	**0.885**	**0.928**

**Table 3:** Ablation study results of FDA and dynamic weight ω on VT821 dataset.

FDA	ω	VT821			
		MAE↓	$F_{m}^{\mathrm{adap}}↑$	$S_{m}↑$	$E_{m}^{\mathrm{adap}}↑$
		0.038	0.823	0.873	0.906
✓		0.031	0.839	0.880	0.929
✓	✓	0.030	0.845	0.885	0.925

**Table 4:** Ablation study results of guidance acquisition methods on VT821 dataset.

Guidance	VT821			
	MAE↓	$F_{m}^{\mathrm{adap}}↑$	$S_{m}↑$	$E_{m}^{\mathrm{adap}}↑$
direct guidance	0.033	0.825	0.873	0.912
$g_{4}$ upsample	0.035	0.828	0.873	0.913
$g_{4}$ upsample + concatenate	0.033	0.831	0.877	0.913
**gradual deep guidance integration**	**0.030**	**0.845**	**0.885**	**0.925**

**Table 5:** MAE, adaptive F-measure, S-measure, and adaptive E-measure comparisons with 11 different RGB-T SOTA methods.

Model	Backbone	VT821				VT1000				VT5000			
		MAE↓	$F_{m}^{\mathrm{adap}}↑$	$S_{m}↑$	$E_{m}^{\mathrm{adap}}↑$	MAE↓	$F_{m}^{\mathrm{adap}}↑$	$S_{m}↑$	$E_{m}^{\mathrm{adap}}↑$	MAE↓	$F_{m}^{\mathrm{adap}}↑$	$S_{m}↑$	$E_{m}^{\mathrm{adap}}↑$
ADFNet [17]	VGG16	0.077	0.717	0.810	0.810	0.034	0.847	0.910	0.921	0.048	0.788	0.864	0.891
CGFNet [21]	VGG16	0.036	0.839	0.879	0.914	0.023	0.895	0.921	0.952	0.035	0.835	0.882	0.924
CSRNet [34]	ESPNetV2	0.037	0.830	0.884	0.911	0.024	0.876	0.918	0.939	0.041	0.810	0.867	0.907
MMNet [20]	VGG19	0.039	0.795	0.875	0.893	0.027	0.861	0.917	0.928	0.043	0.782	0.864	0.890
MIDD [19]	VGG16	0.045	0.804	0.870	0.895	0.027	0.881	0.915	0.942	0.043	0.801	0.867	0.899
ECFFNet [35]	ResNet34	0.034	0.807	0.877	0.907	0.021	0.873	0.923	0.946	0.037	0.803	0.874	0.910
APNet [36]	VGG16	0.034	0.814	0.867	0.911	0.021	0.879	0.921	0.949	0.034	0.816	0.875	0.916
DCNet [37]	VGG16	0.033	0.840	0.877	0.920	0.021	0.900	0.922	0.954	0.035	0.846	0.872	0.924
LSNet [38]	MobileNetV2	0.033	0.822	0.878	0.911	0.022	0.880	0.925	0.950	0.037	0.821	0.877	0.919
TAGFNet [23]	VGG16	0.034	0.821	0.880	0.909	0.021	0.889	0.926	0.951	0.035	0.827	0.883	0.917
MGAI [22]	Res2Net50	0.031	0.828	**0.890**	0.917	0.021	0.885	0.926	0.947	0.034	0.823	0.883	0.918
CFRNet	ResNet34	**0.030**	**0.845**	0.885	**0.925**	**0.020**	**0.900**	**0.926**	**0.955**	**0.032**	**0.846**	**0.885**	**0.928**

Note: ↑ indicates higher values are better; ↓ indicates lower values are better. This notation applies to all similar tables throughout the paper.

## Data Availability

The datasets used in this study are publicly available. The VT821 dataset can be downloaded from http://chenglongli.cn/people/lcl/journals.html, the VT1000 dataset can be downloaded from http://chenglongli.cn/people/lcl/dataset-code.html, and the VT5000 dataset can be downloaded from https://github.com/lz118/RGBT-Salient-Object-Detection.
